# Peptide-MHC Cellular Microarray with Innovative Data Analysis System for Simultaneously Detecting Multiple CD4 T-Cell Responses

**DOI:** 10.1371/journal.pone.0011355

**Published:** 2010-06-28

**Authors:** Xinhui Ge, John A. Gebe, Paul L. Bollyky, Eddie A. James, Junbao Yang, Lawrence J. Stern, William W. Kwok

**Affiliations:** 1 Benaroya Research Institute at Virginia Mason, Seattle, Washington, United States of America; 2 Department of Pathology, Department of Biochemistry and Molecular Pharmacology, University of Massachusetts Medical School, Worcester, Massachusetts, United States of America; University of California San Francisco, United States of America

## Abstract

**Background:**

Peptide:MHC cellular microarrays have been proposed to simultaneously characterize multiple Ag-specific populations of T cells. The practice of studying immune responses to complicated pathogens with this tool demands extensive knowledge of T cell epitopes and the availability of peptide:MHC complexes for array fabrication as well as a specialized data analysis approach for result interpretation.

**Methodology/Principal Findings:**

We co-immobilized peptide:DR0401 complexes, anti-CD28, anti-CD11a and cytokine capture antibodies on the surface of chamber slides to generate a functional array that was able to detect rare Ag-specific T cell populations from previously primed *in vitro* T cell cultures. A novel statistical methodology was also developed to facilitate batch processing of raw array-like data into standardized endpoint scores, which linearly correlated with total Ag-specific T cell inputs. Applying these methods to analyze Influenza A viral antigen-specific T cell responses, we not only revealed the most prominent viral epitopes, but also demonstrated the heterogeneity of anti-viral cellular responses in healthy individuals. Applying these methods to examine the insulin producing beta-cell autoantigen specific T cell responses, we observed little difference between autoimmune diabetic patients and healthy individuals, suggesting a more subtle association between diabetes status and peripheral autoreactive T cells.

**Conclusions/Significance:**

The data analysis system is reliable for T cell specificity and functional testing. Peptide:MHC cellular microarrays can be used to obtain multi-parametric results using limited blood samples in a variety of translational settings.

## Introduction

As important initiators of adaptive immunity, CD4 T cells express highly specific T-cell receptors that recognize epitopes derived from protein antigens in the context of Class II MHC complexes. In the last decade, epitopes associated with a variety of infectious diseases, allergies, tumors and autoimmune diseases have been extensively explored. The advent of the Class II MHC tetramer and its related applications such as tetramer-guided epitope mapping have substantially increased the efficiency and accuracy of the epitope discovery process [1], [2]. As adequate epitope information has been generated, one of the next challenges is to integrate this knowledge to examine overall cellular immune responses in relevant disease settings. Undoubtedly, tetramer technology continues to provide a promising approach for *ex vivo* and *in vitro* Ag-specific T cell analysis. However, technical issues such as the number of fluorochoromes that are distinguishable by current flow cytometry technology limit the simultaneous analysis of multiple antigen specificities. Although the latest peptide:MHC multi-color tetramer staining protocol managed to examine up to 15 Ag-specific T cell populations [3], most often, the detection is restricted to one or two epitopes at a time.

Cellular microarrays provide an alternative solution [4], [5], [6], [7], [8]. This approach uses a high-precision robot arrayer to spot peptide:MHC complex onto a glass slide with high density. Much like artificial antigen presenting cells, the immobilized peptide:MHC activates Ag-specific T cells and triggers cytokine secretion. By co-immobilizing cytokine capture antibody with the peptide:MHC complex on the glass surface, the cytokines secreted by the activated T cells are retained *in situ* and probed with fluorescence-conjugated cytokine detection antibody for quantification. Unlike tetramer staining, which provides superb single-cell-level characteristics, the aim of peptide:MHC cellular microarrays is to achieve a high-throughput detection solution. The collection of various peptide:MHC complexes defines the number of Ag-specificities for evaluation. The cytokine capture antibodies, in turn, define the functional parameters that are measured. By using predetermined spatial coordinates (rather than a panel of fluorescent tags in the flow cytometry setting), a multitude of different functional T cell responses can be distinguished and/or compared in a single assay.

Despite their potential, cellular microarrays are still in their infancy. Previous studies primarily focused on method development using *in vitro* established Ag-specific T cell lines or primary T cells obtained from TCR transgenic mice [4], [7], [8]. Only a single report addressed the changes of tumor antigen-specific CD8 T cell responses due to vaccination [5]. In this study, combining pioneering work with our CD4 T cell epitope identification knowledge, we designed a protocol for functional peptide:MHC microarray production and a data analysis system suitable to evaluate the cytokine responses of primary human CD4 T cells. In the first application, we examined the IFNgamma response of human CD4 T cells against 36 Influenza A derived epitopes. The compiled results provided us with a hierarchy of influenza A epitope specific T cell responses in the general population. This type of knowledge may provide insights for vaccine development in the future. In addition, the subject-by-subject results revealed similarities and differences in anti-viral responses in different individuals. This type of knowledge may be useful for risk prediction or diagnosis in infectious disease related translational research. In the second application we investigated autoimmune Type 1 Diabetes related self-antigen specific CD4 T cell responses. We compared IFNgamma and IL10 responses from T cells from both diabetic and non-diabetic individuals – a typical cross-sectional study for comparative purposes. Among 10 epitopes derived from a panel of putative pancreatic beta-cell self-antigens, there was no single epitope that distinguished diabetic from non-diabetic subjects. In addition, the accumulated IFNgamma or IL10 response was not significantly different between individuals from the two groups.

## Results

### Characterization of the peptide:MHC cellular microarray

**Figure 1:**
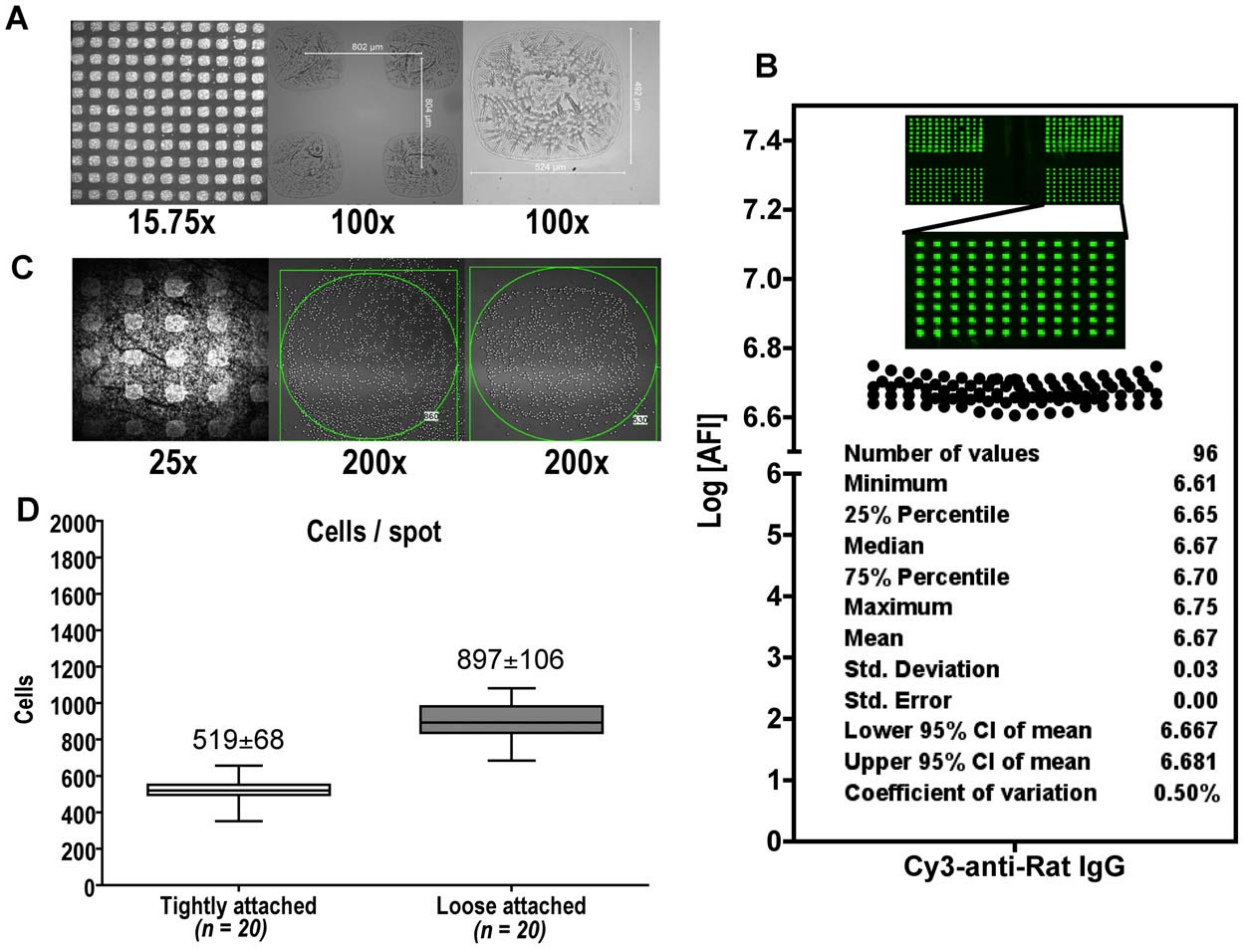
Characteristics of peptide:MHC cellular microarrays. (A) A part of a freshly fabricated microarray image with magnification of 15.75× (left panel), 100× (middle panel) and 200× (right panel). (B) A raw fluorescence image of a 4-supergrid 8×12 microarray probed with Cy3-conjugated anti-Rat-IgG. The absolute fluorescence of all 96 spots for the low-right supergrid was quantified and analyzed for the variance. (C) A microarray loaded with 6×10^6^ primary human CD4 T cells. (Left panel, 25× magnification) image taken immediately after 6 hours of incubation; (middle panel, 200× magnification) image taken after aspirating culture medium; (right panel) image taken after washing the slide with 1xPBS for three times. The numbers at the low-right corners of middle and right panels indicate cell counts. (D) The box/whisker plots represent cell counts of 20 randomly selected spots before (loose attached) and after (tightly attached) washing procedure.

We used a contact-printing robot to fabricate our cellular microarray. The pin repeatedly delivered ∼12.5 nl/spots of sample solution onto the glass surface of a chamber slide in a pre-arranged format (Figure 1A far-left panel), yielding a series of spots with the size ∼0.5 mm×0.5 mm (Figure 1A far-right panel). For a typical 1-chamber slide, the size ratio of printable surface (18×47 mm) to a single spot was approximately 3,400:1. The distance between the centers of two adjacent spots was 0.8 mm (Figure 1A middle panel). The size and amount of proteins immobilized on the surface was highly uniform, with 0.50% co-efficient of variation (Figure 1B). The spot size and the cell density determined the number of cells possibly settling on each spot. Since only the cells “settling down” on the top of a spot had an opportunity to be activated, the number of cells “settling” on top of the spots was theoretically relevant to assay sensitivity. In practice, when we loaded 6×10^6^ cells on a 1-chamber slide, the average number of cells attaching to each individual spot was 897±106 (loose attached) (Figure 1C, left and middle panel; Figure 1D). Around 519±68 cells were firmly attached, even after two rounds of washing with 1xPBS (tightly attached) (Figure 1C, right panel and Figure 1D). Given the fact that at least one single Ag-specific T cell is required to produce cytokine upon activation, based on the cell number/spot as we counted, the best estimate of the detection limit for Ag-specific T cells is as low as 0.1–0.2% among all the cells loaded on the slide. Taken together, by using this setting, we can easily print an array of 20 rows×50 columns in a 1-chamber slide. With 10 replicates for each peptide:MHC/cytokine feature, this format allows us to evaluate up to 100 features, simultaneously. The lowest number of Ag-specific T cells (among those total 6×10^6^ T cells loaded onto the chamber slide) required for generating detectable signal range from 6,000 to 12,000. Increasing the total number of cells input improved the number of cells “landing” on those spots, but it negatively influenced signal/noise ratio of fluorescence measurements.

### Scoring system to normalize Ag-specific cytokine responses from different experiments

**Figure 2:**
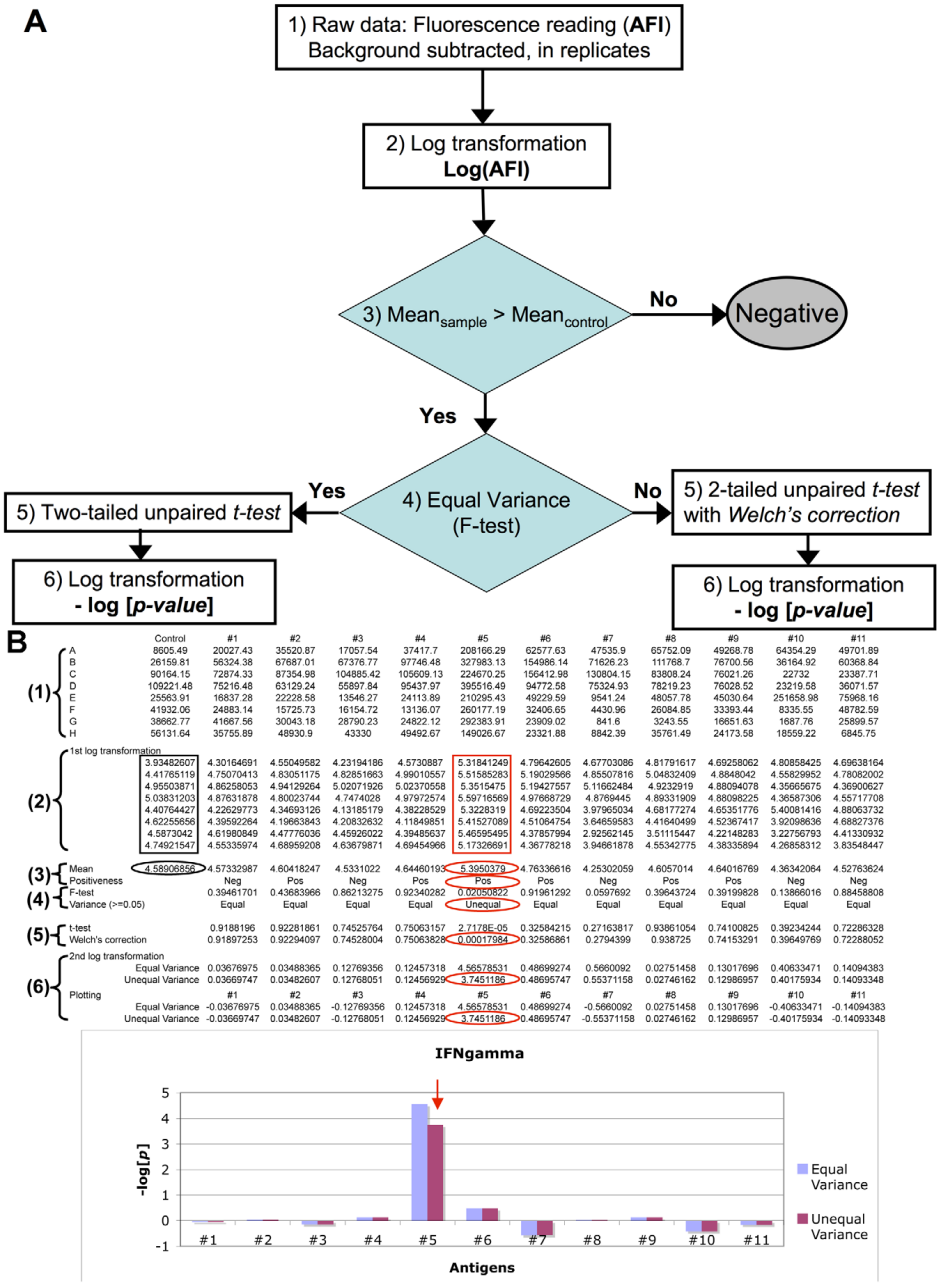
Illustration of data analysis. (A) Flow-chart; (B) a datasheet layout for a 8×12 cellular microarray assay. The contents in black or red boxes/ovals are relevant to generating final result highlighted by red arrow in the bar graph. The numeric indicators in the brackets of (A) and (B) are consistent.

**Figure 3:**
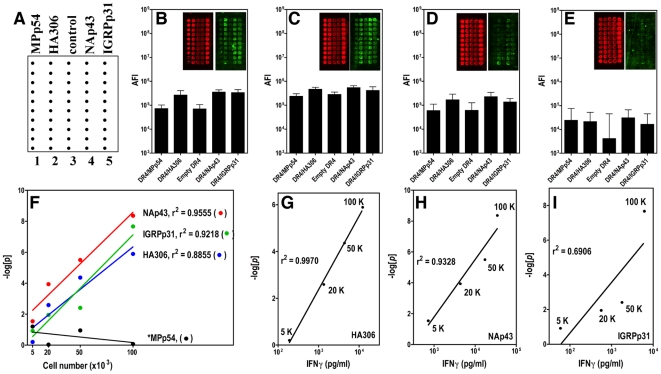
Correlation between microarray results and Ag-specific T cell inputs. A guide to print one 10×5 cellular microarray in one subunit of a 4-chamber slide (A). Raw fluorescence images and measured Absolute Fluorescence Intensity (AFI) of an array chamber loaded with 100×10^3^ (B), 50×10^3^ (C), 20×10^3^ (D) and 5×10^3^ (E) of Ag-specific HA306, NAp43 and IGRPp31-specific CD4 T cells. No MPp54-specific T cells were loaded for assaying. Green fluorescence images on the right of each panel represent Ag-specific cytokine production signals probed by AlexFluor555-conjugated streptavidin while red fluorescence images on the left of each panel represent non-specific signals probed by Cy5-conjugated anti-Rat IgG to facilitate the spot identification. Band-pass filters avoided spectral overlap. Only the green fluorescence was measured as AFI and proceeded for quantification. Correlations between “**−log[** ***p*** **]**” and corresponding numbers of HA306, NAp43 and IGRPp31 specific CD4 T cells are shown in panel (F). MPp54 data in (F) represents Ag non-specific signals revealed by corresponding chip assays. Correlations between “**−log[** ***p*** **]**” and IFNgamma production for HA306 (G), NAp43 (H) and IGRPp31 (I) are also shown to determine the concordance between two different assays (microarray assay and ELISA).

To assess clinical samples using the cellular microarray, particularly for comparative purposes, a standardized data analysis system is required to transform original fluorescence measurements into normalized results suitable for inter-/intra-assay comparison. We designed a 6-step protocol of mathematic/statistical calculations (Figure 2A): 1) the absolute fluorescence intensity (AFI) of each spotted area was measured as raw data; 2) a logarithmic transformation of the raw data was taken; 3) replicates were grouped and compared with the negative control (replicate spots of empty Class II MHC); 4) the variances of experimental replicates and control replicates were compared using an F-test; 5) based on F-test results, one of two types of 2-tailed non-paired *t-tests* was chosen to compare the means of the experimental group and the control group – a regular t-test for equal variances or a t-test with Welch's correction for unequal variances; 6) another logarithmic transformation was performed to convert the *p-value* of the t-test into a more readable score. This analysis system ranks Ag-specific T cell responses by evaluating the statistical difference between the experimental group and a ubiquitous negative reference. In this specialized detection system where no standard curve was available to normalize the activation level of a T cell response and the variation of fluorescence signal was high, the advantage of this data analysis approach was that both the AFI values and the variances (from either a sample or the negative control) contributed to the underlying *p-values* (or **−log[** ***p*** **]** scores). Since all **−log[** ***p*** **]** scores were calculated against a common internal negative control, these scores were independent of the particular assay and could be directly compared. To further validate the biological implication of this scoring method, we compared the cellular microarray results of serially diluted CD4 T cell lines with IFNgamma production in a parallel control experiment, in which the same numbers of T cells were seeded into a 96-well plate coated with peptide:MHC and anti-CD28/CD11a for the same period of stimulation (Figure 3). We found that those AFIs had less indicative value to reveal the number of Ag-specific T cells for the assays from different chamber. However, the **−log[** ***p*** **]** scores were not only correlated with the input of Ag-specific T cells (Figure 3F), but also correlated with IFNgamma production (from the control experiment) measured by conventional ELISA (Figure 3G–I). To facilitate batch data processing, a coding program based on this 6-step procedure was created with MS-Excel so that the **−log[** ***p*** **]** score for various peptide:MHC features could be calculated and reported equivalently (Figure 2B).

### Using peptide:MHC/IFNgamma microarrays to study Influenza A specific T cell responses

**Figure 4:**
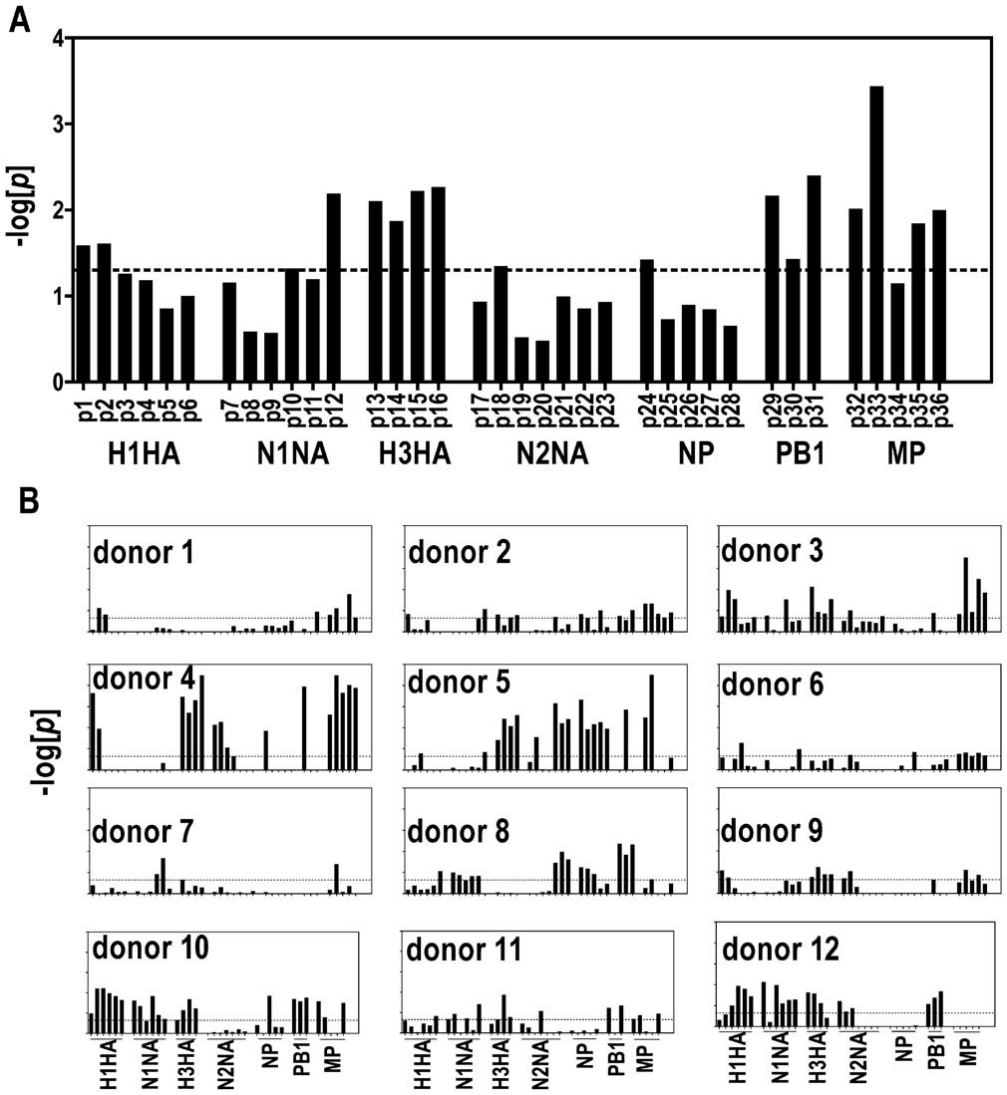
Results of using peptide:MHC/IFNgamma microarray to measure 36 different influenza A specific CD4 T cell responses. (A)The average score for each influenza A peptide (*n = 12*); (B) Individual plot for each DR0401 donor. Dotted lines represent the **−log[** ***p*** **]** score equivalent to 95% confidence limit (*p = 0.05*) to further distinguish ambiguous score (*p>0.05*) from less ambiguous score (*p<0.05*).

Our first application of the peptide:MHC cellular microarray was to study Influenza A specific CD4 T cell responses in randomly selected HLA-DR0401 (DRA1*0401/DRB1*0401) subjects. Purified CD4 T cells were stimulated with the trivalent vaccine *in vitro* in the presence of autologus antigen presenting cells to boost Ag-specific T cell frequencies prior to cellular microarray assays. A total of 36 DR0401-restricted epitopes derived from hemagglutinin (HA), neuraminidase (NA), nuclear protein (NP), polymerase B1 (PB1) and M1 matrix protein (MP) were assessed on a single microarray simultaneously. The overall responses for all 12 subjects indicated that MP, PB1 and H3HA specific responses were relatively dominant over other antigens (Figure 4A and Table 1). Figure 4B summarized the T cell responses from each subject, revealing highly heterogeneous response profiles among this study population. Donors 1, 2, 6, 7, 9 and 11 showed weak responses to this influenza A panel with only a few detectable responses. Donor 3 had strong MP specific responses, moderate H3HA and H1HA responses. Donor 4 had strong MP and H3HA specific responses and moderate responses to H1HA and N2NA. Donor 5 had strong H3HA, N2NA, NP, PB1 and MP specific responses. Donor 8 had moderate responses to N2NA, NP and PB1. Donor 10 had moderate responses to H1HA, N1NA, H3HA and PB1. Donor 12 had moderate responses to H1HA, N1NA, H3HA, N2NA and PB1.

**Table 1:** Summary for Influenza A specific T cell responses.

i.d	Peptide	Sequence	aMean (*n = 12*)	bRanking
p1	H1HA203	203-NQRALYHTENAYVSVVS-219	1.59	13
p2	H1HA328	328-LRMVTGLRNIPSIQSRG-344	1.61	12
p3	H1HA334	334-LRNIPSIQSRGLFGAIA-350	1.26	18
p4	H1HA392	392-TNKVNSVIEKMNTQFTA-408	1.18	20
p5	H1HA398	398-VIEKMNTQFTAVGKEFN-414	0.85	28
p6	H1HA440	440-ELLVLLENERTLDFHDS-456	1.00	23
p7	N1NA96	96-GWAIYTKDNSIRIGSKG-112	1.16	21
p8	N1NA124	124-CSHLECRTFFLTQGALL-140	0.59	33
p9	N1NA219	219-WKKRILRTQESECVCVN-235	0.57	34
p10	N1NA249	249-GAASYKIFKIEKGKVTK-265	1.32	17
p11	N1NA369	369-KGFEMIWDPNGWTDTDS-385	1.19	19
p12	N1NA416	416-DCIRPCFWVELVRGLPR-432	2.19	5
p13	H3HA17	17-HHAVSNGTLVKTITNDQIEV-36	2.10	7
p14	H3HA97	97-CYPYDVPDYASLRSLVASSG-116	1.87	10
p15	H3HA297	297-VNRITYGACPRYVKQNTLKL-316	2.22	4
p16	H3HA305	305-CPRYVKQNTLKLATGMRNVP-324	2.27	3
p17	N2NA48	48-NQVMLCEPTIIERNITE-64	0.94	25
p18	N2NA96	96-GFAPFSKDNSIRLSAGG-112	1.35	16
p19	N2NA206	206-IYNGRLVDSIVSWSKEI-222	0.52	35
p20	N2NA236	236-TCTVVMTDGSASGKADT-252	0.48	36
p21	N2NA260	260-GKIVHTSTLSGSAQHVE-276	1.00	24
p22	N2NA390	390-LQINRQVIVDRGNRSGY-406	0.85	29
p23	N2NA402	402-NRSGYSGIFSVEGKSCI-418	0.93	26
p24	NP73	73-ERRNKYLEEHPSAGKDPKKT-92	1.42	15
p25	NP321	321-NP1HKSQLVWMACHSAAFED-340	0.73	31
p26	NP401	401-ASAGQISIQPTFSVQRNLPF-420	0.90	27
p27	NP433	433-TEGRTSDMRTEIIRMMESAR-452	0.84	30
p28	NP441	441-RTEIIRMMESARPEDVSFQG-460	0.65	32
p29	PB1/34	34-TGTGYTMDTVNRTHQ-48	2.17	6
p30	PB1/281	281-KLANVVRKMMTNSQDTE-297	1.43	14
p31	PB1/410	410-GMFNMLSTVLGVSILNLGQ-428	2.40	2
p32	MP9	9-TYVLSIVPSGPLKAEIAQRL-28	2.02	8
p33	MP57	57-KGILGFVFTLTVPSERGLQR-76	3.44	1
p34	MP73	73-GLQRRRFVQNALNGNGDPNN-92	1.15	22
p35	MP97	97-VKLYRKLKREITFHGAKEIA-116	1.84	11
p36	MP177	177-NRMVLASTTAKAMEQMAGSS-196	2.00	9

a The average of −log[*p*].

b based on mean value of −log[*p*].

### Using peptide:MHC cellular microarrays to monitor T cell responses to beta-cell autoantigens

**Figure 5:**
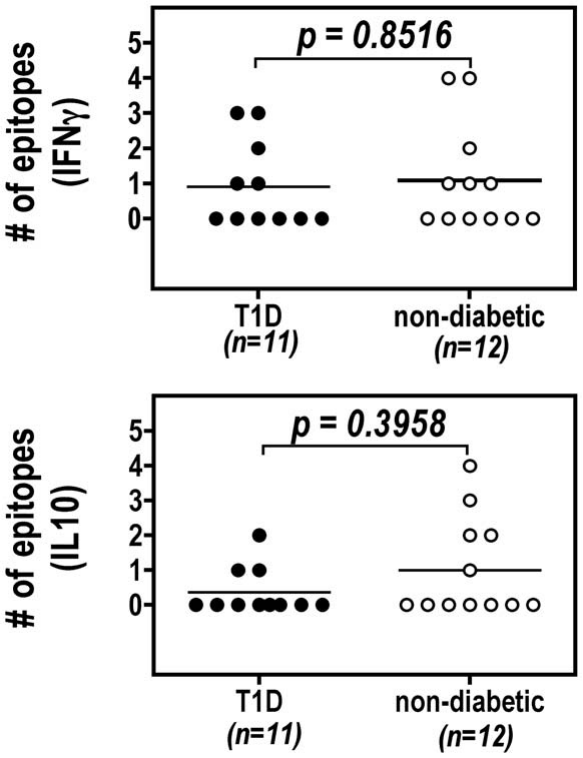
The counts of epitope specific IFNgamma (A) or IL10 (B) response for each individuals. Each circle represents a diabetic (filled circle) or a non-diabetic subject (open circle). A Mann-Whitney test was used for statistical calculation.

A second, more challenging application was to compare self-antigen specific T cell responses in individuals expressing autoimmune Type 1 Diabetes susceptible Class II HLA-DR0401 haplotypes. We selected 10 epitopes derived from putative beta-cell self-antigens [9], [10], [11], [12], [13], [14], [15]. Purified CD4 T cells were stimulated with the candidate peptides in the presence of autologus antigen presenting cells to boost Ag-specific T cell frequencies prior the assays. The IFNgamma and the IL10 responses from T cells specific for the 10 candidate epitopes were investigated at the same time (Table 2). Like other cross-sectional studies, average **−log[** ***p*** **]** scores of T cell responses for T1D patients (n = 11) and non-diabetic individuals (n = 12) were compared (Table 3). Only the GAD65p70-specific IL10 response was significantly different between the two populations (*p = 0.0315*, Mann-Whitney test). The non-diabetic group was associated with higher GAD65p70 specific IL10 responses (0.4483±0.1609) than the T1D group (0.04909±0.04157). However, this difference of GAD65p70-specific IL10 responses between the two groups could be caused by chance alone since the p-value was higher than 0.05 after false discovery rate adjustment. The non-diabetic group was also associated with a trend of increased GAD65p15-specific IL10 responses (0.5817±0.1671) in comparison to the diabetic group (0.1973±0.1107). However, that difference was not statistically significant (*p = 0.0777*, Mann-Whitney test). We also examined the differences of T cell responses in a discontinuous trait fashion using a 95% confidence limit (p<0.05) to distinguish ambiguous results from those less ambiguous results. A positive response was defined as **−log[** ***p*** **]**≥1.30 (p≤0.05, 95% confidence limit), whereas a negative response was defined as **−log[p]**<1.30. A majority of subjects did not elicit any detectable IFNgamma or IL10 responses (Figure 5A–B). No donor responded to more than 4 epitopes. The counts of detectable epitope specific responses for each individual were almost evenly distributed between the diabetic and non-diabetic groups. For each individual epitope, no significant difference (Mann-Whitney Test) was detected between these two groups (Table 4), although there were trends showing that (i) GAD65p70-specific IFNgamma responses were slightly increased in T1D group; (ii) GAD65p15 and GAD65p35-specific IL10 responses were slightly increased in the non-diabetic group. Among all 10 epitopes we studied, GAD65p15 (6/23), PPIp57 (4/23), GAD65p70 (4/23) were associated with the most prevalent IFNgamma response while DMKp2 (5/22), GAD65p35 (4/23) and PPIp57 (3/23) were associated with the most prevalent IL10 responses (by examining the counts in Table 4).

**Table 2:** Putative beta-cell autoantigenic peptides.

i.d	Peptide	Sequence	References
1	aZnT8p28s	271-SILLMEGVPKSLN-283	[10] and unpublished data
2	bPPIp57	73-GAGSLQPLALEGSLQKRGIV-92	[9], [11]
3	cIGRPp3	17-KDYRAYYTFLNFMSNVGDPR-36	[12]
4	IGRPp31	241-KWCANPDWIHIDTTPFAGLV-260	[12]
5	dGAD65p15	113-DVMNILLQYVVKSFDRSTKV-132	[13]
6	GAD65p35	273-LIAFTSEHSHFSLKKGAAAL-292	[13]
7	GAD65p70	553-KVNFFRMVISNPAATHQDID-572	[14]
8	eDMKp2	9-RLQQLVLDPGFLGLEPLLDL-28	unpublished data
9	DMKp23	177-AEIVMAIDSVHRLGYVHRDI-196	unpublished data
10	fIA4p46	953-VRSKDQFEFALTAVAEEVNA-972	[15]

a Zinc transporter.

b Preproinsulin.

c Islet-specific gluose-6-phosphatase catalytic subunit-related protein.

d Glutamic Acid Decarboxylase 65.

e Dystrophia Myotonica Kinase.

f Islet protein tyrosine phosphates.

**Table 3:** Beta-cell epitope specific T cell responses.

Epitope	Cytokine	aMean ± SD		b *P*	c *P (FDR.adj)*
		T1D (*n = 11*)	non-T1D (*n = 12*)		
**ZnT8p28s**	**IFNgamma**	0.1973±0.1013	0.1900±0.1537	0.5524	0.7158
	**IL10**	0.2082±0.1113	0.0400±0.03075	0.1423	0.3840
**PPIp57**	**IFNgamma**	0.4427±0.2308	0.4417±0.2633	0.9753	1.0000
	**IL10**	0.3136±0.1326	0.1917±0.1351	0.4556	0.7158
**IGRPp3**	**IFNgamma**	0.6136±0.2161	0.3008±0.1619	0.1536	0.3840
	**IL10**	0.1136±0.03681	*0.1445±0.1367	0.0845	0.3840
**IGRPp31**	**IFNgamma**	0.0200±0.01401	0.3875±0.2114	0.1221	0.3840
	**IL10**	0.1382±0.08814	0.3200±0.1796	0.5494	0.7158
**GAD65p15**	**IFNgamma**	1.193±0.4842	1.482±0.6396	0.7345	0.8641
	**IL10**	0.1973±0.1107	0.5817±0.1671	0.0777	0.3840
**GAD65p35**	**IFNgamma**	0.3800±0.1984	0.2450±0.1907	0.2534	0.5631
	**IL10**	0.3764±0.1684	0.6675±0.2513	0.5140	0.7158
**GAD65p70**	**IFNgamma**	0.5527±0.2052	0.7283±0.4938	0.3684	0.7158
	**IL10**	0.04909±0.04157	0.4483±0.1609	***0.0315***	0.3840
**DMKp2**	**IFNgamma**	0.5464±0.1413	0.4250±0.2915	0.1290	0.3840
	**IL10**	0.5091±0.1988	*0.9891±0.2402	0.1226	0.3840
**DMKp23**	**IFNgamma**	0.06909±0.03781	0.2550±0.1820	0.8020	0.8911
	**IL10**	0.0700±0.03057	0.2817±0.1288	0.5726	0.7158
**IA2p46**	**IFNgamma**	0.05545±0.02745	0.1108±0.09193	0.5482	0.7158
	**IL10**	0.1791±0.08849	0.1333±0.06713	1.0000	1.0000

a The average of −log[*p*].

b *P* was calculated by 2-tailed Mann-Whitney test.

c *P* was calculated with false discovery rate (FDR) adjustment using the Benjamini-Hochberg correction.

*only 11 non-T1D subjects were investigated.

**Table 4:** Beta-cell epitope specific T cell responses with 95% confidence correction (−log[*p*]≥1.30).

	IFNgamma					IL10				
	T1D (n = 11)		Non-T1D (n = 12)		a *P*	T1D (n = 11)		Non-T1D (n = 12)		a *P*
	bPositive	cNegative	bPositive	cNegative		bPositive	cNegative	bPositive	cNegative	
**ZnT8p28s**	0	11	1	11	1	0	11	0	12	1
**PPIp57**	2	9	2	10	1	1	10	2	10	1
*IGRPp3	1	10	1	11	1	0	11	1	10	1
**IGRPp31**	0	11	1	11	1	0	11	1	11	1
**GAD65p15**	3	8	3	9	1	0	11	2	10	0.4783
**GAD65p35**	1	10	1	11	1	1	10	3	9	0.5901
**GAD65p70**	2	9	1	11	0.59	0	11	1	11	1
*DMKp2	1	10	1	11	1	2	9	3	8	1
**DMKp23**	0	11	1	11	1	0	11	0	12	1
**IA2p46**	0	11	0	12	1	0	11	0	12	1

a *P*-value was calculated by 2-tailed Fisher Exact test.

b “positive” was defined as a response with −log[*p*]≥1.30, which represents 95% confidence.

c “negative” was defined as a response when −log[*p*]<1.30.

*only 11 non-T1D subjects were investigated.

## Discussion

In this study, we developed a cellular microarray using Class II MHC protein loaded with antigenic peptides. This method combines the highly Ag-specific features of tetramer technology with the advantages of high-throughput measurement offered by microarray platform. By investigating more epitopes and shifting the targets from well-established Ag-specific T cell lines to highly heterogeneous clinical samples, our aim was to adapt, simplify and standardize this peptide:MHC cellular microarray system for translational studies.

For assay development, our first emphasis was to use relatively large size spots to maximize the detection sensitivity even though this practice inevitably diminished the spotting density. The dimension of the spotting pins as well as composition and concentration of spotting reagents were major factors determining the spot size. In our system, the concentrations of peptide:MHC complex and cytokine capture antibody saturated the absorption capacity of the glass surface. By doing so, the condition provided maximal stimuli and cytokine capturing ability. The theoretical detection limit (0.1∼0.2%) that we estimated is consistent with previous discussion by Brown and colleagues [4], [16]. In practice, however, if the cytokine production by the T cells with a certain Ag-specificity is lower than others, a higher number of responding T cells is required. The purpose of the cellular microarray is to determine (or semi-quantify) the presence of Ag-specific T cells rather than providing an exact frequency. Based on its theoretical detection limit, the sensitivity of this assay system is lower than ELISPOT assays and may not be sufficient to detect low frequency or low avidity T cell responses. However, the system should be adequate to detect high frequency or high avidity responses. Second, to answer the call for transparency in data documentation [17], [18], we developed a scoring system based on our understanding of what the system was designed to reveal and what the cellular microarray system was measuring. This data analysis system provided a statistical indication of differences between the group of spots for target epitopes and a baseline. Conceptually, a strong response would associate with high AFI for all spot replicates and low variance between replicates, while a weak response would generate the opposite outcome. This feature would be well reflected by our scoring system - either increasing the overall AFI or decreasing the variance within the same group favored a **−log[** ***p*** **]** score (or a low *p*-value).

We then applied the cellular microarray system to detect immune responses against foreign antigens and self-antigens. Very different results were revealed in these two different types of immunological settings. For foreign antigens, which normally trigger robust adaptive responses, the cellular microarray system performed quite well. Average scores for 17 out of 36 epitopes (47%) were above the 95% confidence limit (*p* = 0.05 or **−log[** ***p*** **]** = 1.30). Among these 17 epitopes, MP, PB1 and H3HA specific responses were more prevalent than others. These more robust Ag-specific responses are likely due to either repeated vaccination or natural infection. It is noteworthy that prior to the outbreak of swine-origin H1N1 influenza A virus (S-OIV) pandemic, H3N2 has been the most dominant circulating subtype in North America for decades. Prevalent responses to H3HA not only imply strong antigenicity of this viral surface antigen, but also broad memory T cell response in the general population. In addition, MP and PB1 are internal antigens and the most conserved components among different strains and even subtypes. Many types of influenza A strains (H1N1, H3N2, H5N1) can boost memory responses specific to these epitopes. The relatively weak H1HA specific responses might indicate a call for new vaccines for emerging H1N1 threats, such as the Swine-origin H1N1 outbreak in 2009 [19], [20].

In the autoimmune disease setting, the performance of the cellular microarray system was less impressive. Overall, the measured microarray responses were weak. Among the list of 10 putative autoantigenic epitopes, only GAD65p70 elicited stronger IL10 responses in the non-diabetic group than in the diabetic group. Although that observation is consistent with a previous notion that self-reactive CD4 T cells in non-diabetic subjects might confer protection by producing regulatory cytokines such as IL10 while those cells in diabetic groups could not [9], the average scores (**−log[** ***p*** **]** = 0.4483±0.1609 for non-T1D group vs. 0.04909±0.04157 for T1D group) for both populations were lower than our 95% confidence limit. Despite being statistically significant by the Mann-Whitney test, the p-value was higher than 0.05 after false discovery rate adjustment. This indicated that the difference of GAD65p70-specific IL10 responses between two study groups could be due to chance alone. For the autoimmune diabetes study, interpreting the data in a discontinuous trait fashion (using 95% confidence limit as a cutoff) seemed to be more appropriate since the majority of responses were weak or null. Based on this consideration, we concluded that there were no detectable differences between any of these 10 epitope specific CD4 T cells in peripheral blood of diabetic and non-diabetic individuals as measured by the cellular microarray. This indicated that assessing T cell responses against these epitopes using the cellular microarray did not provide a prediction of autoimmune status or progression. Clearly, the low detection sensitivity of this assay certainly contributed to the difficulty in detecting differences in the T cell responses of diabetic and non-diabetic subjects. It is also possible that the frequencies of self-reactive T cells might peak near the time that disease is diagnosed and decrease afterwards so that autoreactive T cells in long-term T1D patients (like those recruited for this study) could decrease to levels that are only subtly different from non-T1D subjects and difficult to distinguish regardless of the assay used. In addition, the distribution of autoreactive T cells in peripheral blood and other secondary lymph organs such as pancreatic lymph nodes may not be equivalent. Based on our results, conclude that further refinements are needed to apply the cellular microarray to measure self-reactive T cell responses in peripheral blood.

While progress has been made, there are still challenges for cellular microarrays. First, the procedure for fabricating high quality arrays is still technically difficult without sophisticated equipment and high quality printing substrates. Second, unlike DNA arrays, in which no secondary and/or tertiary structure is required for a nuclear acid probe (immobilized on a solid surface) to maintain its function and specificity, most proteins spotted on the solid surface do no maintain their function over time. In fact, the performance of cytokine capture antibodies from different vendors varied dramatically even under the same fabrication condition. Optimizing the stability of functional proteins is essential to batch produce cellular microarrays that can provide consistent results. A recent study using complementary DNA tags as adaptors to immobilize peptide:MHC on the slides is innovative [16]. Because the protein components are anchored on a slide through various DNA adapters, changes of functional conformation due to direct physical adsorption are avoided. In addition, since proteins are cross-linked to the immobilized cDNA just prior to the assay, they are not required for the spotting procedure. Therefore, this approach decreases the overall duration that protein is exposed on the solid surface.

In summary, we herein describe fabrication and data interpretation procedures for multiplex cellular microarray analysis of Ag-specific T cels. Both the improvements over prior systems and current technical limitations were discussed. The knowledge and experience accumulated through two different applications provided a guide for further refinement of the technology and extending to other disease related translational studies.

## Materials and Methods

### Vaccine, Peptides and antibodies

Fluzone seasonal influenza vaccine (2008–2009 formula) was obtained from Aventis Pasteur (Swiftwater, PA). It contains components of A/Brisbane/59/2007 (H1N1), A/Uruguay/716/2007 (H3N2) and B/Florida/04/2006. In addition to HA and NA, the presence of internal antigens NP, M1MP and PB1 was adequately demonstrated in one of our previous studies [21]. After extensive dialysis to remove nonionic surfactant and preservative, the vaccine was diluted into 1xPBS with protein concentration adjusted to 500 ug/ml. Peptides were purchased from Sigma (St. Louis, MO) or Mimotope (Clayton Victoria, Australia). Functional grade anti-CD28 and anti-CD11a was purchased from eBioscience (San Diego, CA) and Leinco (St. Louis, MO), respectively. For microarray preparation, anti-IFNgamma capture and biotin-labeled detection antibodies were obtained from Pierce (Rockford, IL). Anti-IL10 capture and biotin-labeled detection antibodies were purchased from BD Bioscience (San Diego, CA). IFNgamma ELISA antibody pairs were purchased from eBioscience. Cy3- and Cy5-conjugated donkey anti-mouse IgG, anti-Rat IgG antibodies were purchase from Jackson ImmunoResearch Laboratories (West Grove, PA). AlexFluor555- and AlexFluor647-conjugated streptavidin were obtained from Invitrogen (Carlsbad, CA).

### Subjects

For studying Influenza A specific T cell responses, HLA-DR0401^+^ donors (n = 12) were recruited between June and October 2009. For the diabetes related self-antigen specific T cell study, samples (n = 23) were obtained from both frozen PBMCs and freshly collected peripheral blood from donors recruited between October 2008 and June 2009. The studies were approved by the Institutional Review Board of Benaroya Research Institute (BRI, Seattle, WA). HLA typing was conducted by BRI sequencing and genotyping core facilities. All DR0401^+^ subjects were volunteers of Caucasian descent and were recruited with written consent for these studies.

### Expression, purification of recombinant HLA-DR0401 and Tetramer preparation

Recombinant HLA-DR0401 was expressed by stable transfected S2 cells and purified by affinity chromatography as previously described [1]. To prepare these reagents for arrays, non-biotinylated DR0401 was loaded with candidate peptide in pH6.0 phosphate buffer in the presence of 2.5 mg/ml of *n*-Octyl-D-glucopyranoside (Sigma) and protease inhibitors at 37°C for 72 hours. Peptide:DR4 was thoroughly exchanged into pH7.0 1xPBS using Microcon YM-10 (Millipore, Billerica, MA). Unbound peptide and *n*-Octyl-D-glucopyranoside were also removed during the dialysis. For tetramer, the purified DR0401 was biotinylated according to manufacturer's instruction (Avidity, Aurora, CO). Peptide was loaded as described above and conjugated with PE-streptavidin (Invitrogen).

### Preparation of peptide:MHC Class II cellular arrays

The spotting cocktails contained 100 ug/ml peptide:DR0401, 200 ug/ml cytokine capture antibody, 50 ug/ml anti-CD28 and 50 ug/ml anti-CD11a in pH7.0 1xPBS. The reagents were loaded into a 384-well polypropylene plate and dispensed onto CCL2 chamber slides (FisherScientific, Pittsburgh, PA) with RoboArrayer (BioRad, Hercules, CA) in contact spotting fashion using one single 946MP15XB stealth pin (ArrayIt, Sunnyvale, CA) under 24°C, 60% humidity chamber. The pin dispensed 8–10 replicates for every load of reagent. The pin was rinsed with sonication and dried extensively between sample switching. Slides were stored in a desiccator at 4°C until use.

### Peptide:MHC cellular microarray assaying

Slides were blocked by 2% normal donkey serum (Jackson ImmunoResearch) for 20 minutes at 4°C and rinsed gently by cold 1xPBS. Cultured CD4 T cells (3×10^6^ cells/chamber for 2-chamber slides or 6×10^6^ cells/chamber for 1-chamber slides) were loaded onto peptide:MHC microarray and incubated at a well-leveled 37°C 5% CO_2_ incubator for 6 hours. After incubation, the slides were sequentially washed by 2 cycles of 1xPBS, 2 cycles of H_2_O (5 minutes/cycle to ensure the disruption of the cell) and 2 cycles of washing buffer (1 minute/cycle) containing 1xPBS, 0.02% Tween-20. Slides were then incubated with 1∶250 diluted biotin-labeled detection antibodies at room temperature for 1 hour. Following two cycles of washing, the slides were probed with 1∶1000 diluted AlexFluor555-streptavidin and Cy5-anti-mouse-IgG (or anti-Rat-IgG) for 1 hour at room temperature in the dark then rinsed with washing buffer twice and H_2_O once. Filtered, compressed air was used to remove any liquid remaining on the slides prior to image acquisition.

### Data acquisition, processing and interpretation

Slides were scanned using a Typhoon 9410 Variable Mode Imager (GE Healthcare) under fluorescence acquisition mode. For Cy3- or AlexFluor555 probes, a 532 nm excitation laser source and 580BP30 emission filter were chosen while PMT voltage was set at 750. For Cy5- or AlexFluor647 probes, a 633 nm excitation laser source and 670BP30 emission filter were chosen while the PMT voltage was set at 600. For scan resolution, pixel size was set as 50 µm. The image was initially analyzed using ImageQuant TL v7.0 (GE Healthcare) under Array Analysis section for absolute fluorescence intensity (AFI) measurement. The electronic noise signals (size <3) were filtered. For each slide, the image from a reference channel (probed by Cy5-conjugated anti-mouse and/or anti-Rat IgG) was used to set a grid that positioned the location of individual spot. Manual adjustment was required occasionally. After the grid was properly positioned, image was switched to the sample channel (probed by AlexFluor555-conjugated streptavidin) and fluorescence readings for each spot were recorded. The raw data was exported into MS-Excel for storage and further analysis using the program further described in **Result** section).

### Microscopy and cell counting

After 6 hours of incubation, with the chamber still at mounted position, the slides were gently washed by 1xPBS and observed by using a Leica DM-IRM inverted microscope. Images of individual spots were obtained by using a SPOT RT camera with SPOT-Advance software (Diagnostic Instruments, Serling Heights, MI). The cell number for individuals spots were counted by using ImageQuant TL software under Colony Counting sector.

### Ag-specific CD4 T cell line generation and maintenance

CD4 T cell lines specific to Influenza A Hemagglutinin (HA306: PKYVKQNTLKLAT), Neuraminidase (NAp43: GAASYKIFKIEKGKVTK) and Islet-Specific Glucose-6-Phosphatase Catalytic Subunit-Related Protein (IGRPp31: KWCANPDWIHIDTTPFAGLV) were generated by cell sorting based on positive tetramer staining of T cells from a day-14 *in vitro* peptide stimulated T cell culture. The sorted T cells were maintained in regular T cell culture medium and restimulated with 1ug/ml PHA weekly in the presence of 50 Gy irradiated allogenetic PBMCs and 10 IU/ml of IL2. The purity of the Ag-specific T cells was determined by tetramer staining.

### *In vitro* T cell stimulation

CD4 T cells were purified and stimulated with candidate peptides or viral antigens prepare from vaccine for one week and expanded with recombinant IL2 for another week as previously described [12], [21].

### Cytokine production and ELISA

A flat-bottom 96-well plate was coated with a mixture of 10 ug/ml peptide:DR0401 monomer, 5 ug/ml anti-CD28 and 5 ug/ml anti-CD11a overnight at 4°C. On the next day, the plate was washed once with 1xPBS and seeded with 100×10^3^, 50×10^3^, 20×10^3^ and 5×10^3^ of T cells, respectively. After a brief centrifuge spin to settle down cells, the plate was incubated at 37°C for 6 hours. The concentration of IFNgamma in the supernatant was determined by ELISA using Europium detection system (Perkin Elmer, Waltham, MA).

### Statistical analysis

The coefficient of variation for spotting was estimated using Prism software (GraphPad, San Diego, CA). Differences in Ag-specific T cell responses between T1D and non-diabetic subjects were evaluated with Mann-Whitney test (provided by Prism software) and Fisher's Exact Test (www.langsrud.com/fisher.htm) as *2-tailed p-values*. The Benjamini-Hochberg correction was adapted to calculate *p-values* with false discovery rate (FDR) adjustment.
